# Blockade of adenosine A2A receptor enhances CD8^+^ T cells response and decreases regulatory T cells in head and neck squamous cell carcinoma

**DOI:** 10.1186/s12943-017-0665-0

**Published:** 2017-06-07

**Authors:** Si-Rui Ma, Wei-Wei Deng, Jian-Feng Liu, Liang Mao, Guang-Tao Yu, Lin-Lin Bu, Ashok B. Kulkarni, Wen-Feng Zhang, Zhi-Jun Sun

**Affiliations:** 1 The State Key Laboratory Breeding Base of Basic Science of Stomatology (Hubei-MOST) & Key Laboratory of Oral Biomedicine Ministry of Education, School and Hospital of Stomatology, Wuhan University, 237 Luoyu Road, Wuhan, Hubei Province People’s Republic of China 430079; 2 Department of Oral Maxillofacial-Head Neck Oncology, School and Hospital of Stomatology, Wuhan University, 237 Luoyu Road, Wuhan, 430079 People’s Republic of China; 3 Functional Genomics Section, Laboratory of Cell and Developmental Biology, National Institute of Dental and Craniofacial Research, National Institutes of Health, 9000 Rockville Pike, Bethesda, MD USA

**Keywords:** Adenosine A2A receptor, Head and neck squamous cell carcinoma, Immunotherapy, Regulatory T cells, Anti-tumor response

## Abstract

**Background:**

Cancer immunotherapy offers a promising approach in cancer treatment. The adenosine A2A receptor (A2AR) could protect cancerous tissues from immune clearance via inhibiting T cells response. To date, the role of A2AR in head and neck squamous cell carcinoma (HNSCC) has not been investigated. Here, we sought to explore the expression and immunotherapeutic value of A2AR blockade in HNSCC.

**Methods:**

The expression of A2AR was evaluated by immunostaining in 43 normal mucosae, 48 dysplasia and 165 primary HNSCC tissues. The immunotherapeutic value of A2AR blockade was assessed in vivo in genetically defined immunocompetent HNSCC mouse model.

**Results:**

Immunostaining of HNSCC tissue samples revealed that increased expression of A2AR on tumor infiltrating immune cells correlated with advanced pathological grade, larger tumor size and positive lymph node status. Elevated A2AR expression was also detected in recurrent HNSCC and HNSCC tissues with induction chemotherapy. The expression of A2AR was found to be significantly correlated with HIF-1α, CD73, CD8 and Foxp3. Furthermore, the increased population of CD4^+^Foxp3^+^ regulatory T cells (Tregs), which partially expressed A2AR, was observed in an immunocompetent mouse model that spontaneously develops HNSCC. Pharmacological blockade of A2AR by SCH58261 delayed the tumor growth in the HNSCC mouse model. Meanwhile, A2AR blockade significantly reduced the population of CD4^+^ Foxp3^+^ Tregs and enhanced the anti-tumor response of CD8^+^ T cells.

**Conclusions:**

These results offer a preclinical proof for the administration of A2AR inhibitor on prophylactic experimental therapy of HNSCC and suggest that A2AR blockade can be a potential novel strategy for HNSCC immunotherapy.

**Electronic supplementary material:**

The online version of this article (doi:10.1186/s12943-017-0665-0) contains supplementary material, which is available to authorized users.

## Background

Head and neck squamous cell carcinoma (HNSCC), arising in the oral cavity, larynx, hypopharynx and oropharynx, is the 6th most common cancer worldwide [1]. Despite the intense research interest and efforts in developing therapeutic approaches based on surgical intervention, chemotherapy, radiotherapy, and monoclonal antibody-based therapy, the overall survival rate of HNSCC patients has barely improved [2]. Currently, only 50%–60% of patients can survive 5 years after diagnosis [3]. Generally, smoking, alcohol consumption, and human papillomavirus (HPV) infections are the main risk factors of HNSCC [3]. Notably, HNSCC has been recently identified as an immunosuppressive disease with exhausted T cells, poor antigen-presenting function and accumulation of immunosuppressive cells consisting myeloid-derived suppressor cells (MDSCs), tumor-associated macrophages (TAMs) and regulatory T cells (Tregs) [4, 5]. Therefore, a greater understanding of immunosuppression during the initiation and progression of HNSCC will be valuable to formulate improved therapies.

Solid tumors are often infiltrated by immune cells, predominantly T lymphocytes and myeloid cells [6]. More T cells infiltrating the tumor microenvironment has always been considered as a sign of better prognosis in head and neck cancer [7]. However, a subpopulation of these T cells recruited to human solid tumors are immunosuppressive CD4^+^ Foxp3^+^ regulatory T cells (Tregs) [8]. As compared with the healthy controls, increased accumulation of Tregs was reported in tumor site and peripheral blood of patients with cancer, including HNSCC [9]. Moreover, increased frequency of Tregs has been shown to be closely associated with the poor clinical outcome, presumably due to Tregs-mediated suppression of anti-tumor immunity [10, 11]. Tregs exerts its immunosuppressive functions through distinct and often unexpected mechanism. For instance, Tregs interfere with effector T cell proliferation and cytokine production, inhibit the maturation of antigen presenting cells (APCs) and produce immune suppressive cytokines, such as IL-10 [12, 13]. Therefore, management of Tregs in tumor microenvironment has been underlined as a potential anti-tumor strategy [14].

Immunosuppressive adenosine 3′5’-monophosphate (cAMP)-mediated pathway, signaling through adenosine A2A receptor (A2AR), can inhibit T lymphocytes and natural killer (NK) cells in hypoxic, inflamed, and cancerous microenvironment [15]. A2AR interferes with the trafficking and activities of T cells and NK cells due to the heterologous desensitization of chemokine receptors and reduction in the levels of pro-inflammatory cytokines [15–17]. In the mice that are genetically deficient in A2AR, or in the presence of A2AR specific antagonists, an enhanced T cell- and NK cell-associated tumor rejection was observed [15, 18–20]. In addition, blocking the adenosine-generating pathway CD39/CD73 also induced potent regression of breast cancer, colorectal cancer and melanoma [21–24]. A recent study revealed that A2AR stimulation by agonist in vitro expanded CD4^+^ Foxp3^+^ cells [25]. These researches revealed the potential relevance between adenosine pathway and Tregs. However, to date, the expression and function of A2AR in HNSCC are far from clear.

In the present study, we aimed to identify the correlation between A2AR expression and clinicopathological characteristics in HNSCC tissue microarrays. In vivo, we sought to investigate the anti-tumor effect induced by A2AR pharmacological blockade in genetically defined immunocompetent HNSCC mouse model.

## Methods

### Human tissue samples

Human tissue microarrays (TMAs) include 43 histologically confirmed normal oral mucosae, 48 dysplasia (Dys), 165 primary HNSCC (PH), 12 recurrent HNSCC, and 17 HNSCC with induction chemotherapy (cisplatin, docetaxel and fluorouracil, TPF). HPV infection was determined by immunohistochemistry staining of p16 and further confirmed by HPV DNA in situ hybridization as previously described [26]. The patients received 2 rounds of TPF therapy in accordance with the protocol of Zhang’s clinical trial [27]. Clinical stages of HNSCC patients were classified according to the guidelines of the UICC (International Union Against Cancer) 2002. Pathological grade was determined according to the scheme of WHO (World Health Organization). All the tissues were obtained from the Department of Oral and Maxillofacial Surgery, School and Hospital of Stomatology Wuhan University with the approval of Wuhan University Medical Ethics Committee. The informed consents were obtained from the patients before surgery.

### Mice

The time inducible tissue-specific *Tgfbr1*/*Pten* double conditional knockout (2cKO) mice (*Tgfbr1* ^flox/flox^; *Pten* ^flox/flox^; *K14-CreER* ^tam+/−^) were bred as previously described [28]. The mice maintained and genotyped as previously reported and *Tgfbr1* ^flox/flox^/*Pten* ^flox/flox^ mice (*Tgfbr1* ^flox/flox^; *Pten* ^flox/flox^; *K14-CreER* ^tam−/−^) from same cage were separated as wide type (WT) control. All the mice were bred in the FVBN/CD1/129/C57 mixed background.

### In vivo SCH58261 treatment

The *Tgfbr1/Pten* 2cKO mice were given tamoxifen by oral gavage for 5 consequent days [28]. And these mice randomly divided into two groups including vehicle group (DMSO diluted in PBS, *n* = 6) and A2AR antagonist SCH58261 treatment group (*n* = 6). A week later, SCH58261 (1 mg/kg) or vehicle was intraperitoneally injected into *Tgfbr1/Pten* 2cKO mice every other day until the end point. The endpoint was determined according to a systematic evaluation by the veterinarian. Photographs of tumor-bearing mice were taken at day 19 and day 34. Body weight and the tumor volumes were measured every other day. All mice were euthanized at the end of the study_._

### Flow cytometry

Single cell suspension was isolated from spleen, lymph nodes, peripheral blood and tumors according to a standardized protocol [29]. Cells from different groups including wild type (WT) mice and 2cKO mice in vehicle group or SCH58261 treated group were re-suspended in staining buffer (PBS with 2% FBS) at 4 °C and non-specific Fc was blocked for 10 min. Fluorochrome-conjugated monoclonal antibodies were used for staining: isotype-matched IgG controls, Percp-Cy5.5-conjugated F4/80; PE-conjugated CD11b, IFN-γ; PE-Cy5-conjugated Foxp3, FITC-conjugated CD4, CD8 and Gr1 (eBioscience), Adenosine A2A-R Antibody Alexa Fluor® 647 (Santa Cruz Biotech). For IFN-γ staining, cells were processed with Cell Stimulation Cocktail (plus protein transport inhibitors, eBioscience), which contains Phorbol-12-myristate-13-acetate (PMA), ionomycin, Brefeldin A and Monensin for 12 h following the manufacture’s instruction. Dead cells were excluded by staining 7AAD (Invitrogen). Isotype control and positive control were set for each antibody and each experiment. Different gating strategy was used to identify the cell populations. Data were analyzed with Flowjo 7.6 (Tree Star).

### Isolation of CD8^+^ T cells

CD8^+^ T cells were purified from freshly isolated tumor infiltrated lymphocytes of the 2cKO mice from vehicle group or SCH58261 treated group by immunomagnetic sorting using the mouse CD8^+^ T cell isolation kit and following the manufacturer’s instructions (Miltenyi Biotech). The purity of the isolated CD8^+^ T cells was measured by surface staining with anti-CD8 mAb. The overall purity of the resulting cells was 85.3% ± 1.2%. Cell viability was >90% as measured by trypan blue exclusion.

### Cytokine measurement

Freshly isolated CD8^+^ T cells were cultured in RPMI medium at a concentration of 1 × 10^6^ for 8 h. The supernatants were collected for IFN-γ and TNF-α measurement. The levels of IFN-γ and TNF-α were determined by enzyme-linked immunosorbent assay (ELISA) (BD Pharmingen and R&D System).

### Immunofluorescence

Briefly, the human HNSCC tissue sections were hydrated and antigen retrieval. Then sections were blocked with goat serum and incubated with rabbit polyclonal antibody against A2AR (Abcam) at 4 °C overnight, followed by incubation with fluorochrome conjugated secondary antibodies (Alexa 594 anti-rabbit; Invitrogen) and DPAI (Vector Laboratories). The images were observed and taken using C2+ confocal microscope system (Nikon).

### Immunohistochemistry

Paraffin sections of human HNSCC tissue microarrays or mouse HNSCC section were rehydration in graded alcohol. The antigen retrieval was performed in boiled sodium citrate. All the sections were incubated in 3% hydrogen peroxide for endogenous peroxidase blockade. Goat serum or rodent block (for mouse section) was used to block the non-specific binding at 37 °C for 1 h. Next, sections were incubated with antibody for A2AR (Abcam 1:200), HIF-1α (Abcam 1:200), CD73 (Genetex 1:200), Foxp3 (Abcam 1:100), CD8 (ZSGB-BIO 1:100, for human samples), CD8α (Novus, 1:200, for mouse samples) at 4 °C for 12 h. On the day 2, sections were incubated with secondary biotinylated immunoglobulin G antibody solution and an avidin-biotin-peroxidase reagent. Then, the section stained with DAB kit (Mxb Bio) and the sections lightly counterstained with haematoxylin (Invitrogen, USA). Negative control with primary antibody replaced by PBS, isotype control and commercial available positive control for each antibody were set in parallel.

### Western blot

The mouse tumor tissues were carefully dissected (*n* = 6, respectively) and stored at −80 °C. All samples were lysed with RIPA Lysis Buffer (Beyotime), which contains protease inhibitors and phosphatase inhibitors. Lysates were denatured in loading buffer (Beyotime) at 95 °C for 5 min. Protein samples were separated by 12% SDS-polyacrylamide gel electrophoresis and transferred to polyvinylidene fluoride membranes (Millipore). Rabbit A2AR antibody (Abcam, 1:1000), Rabbit CD73 antibody (Genetex, 1:1000) and Rabbit HIF-1α antibody (Abcam 1:1000) were used as primary antibody, GAPDH (Abcam, 1:3000) was used as loading control. Western blot staining was performed by using enhanced chemiluminescence detection kit (Advansta). All Western blots were repeated at least 3 times.

### Scoring system

The scanning of all slices was performed by Aperio ScanScope CS scanner (Leica, USA) with background subtraction. The histoscore of each slice was quantified by Aperio Quantification System (Version 9.1). The histoscore of pixel quantification was calculated as the total intensity/total cell member.

### Statistical analysis

Statistical analysis was performed GraphPad Prism 5.0 (Graph Pad Software Inc). One-way ANOVO followed Tukey test was applied to analyze the difference in A2AR expression in normal oral mucosa, oral epithelial dysplasia and HNSCC, pathological grades, the population change of CD8^+^ T cells, MDSCs and TAMs in spleen, lymph nodes and peripheral blood of different groups and the spleen index. Unpaired *t* test was used to analyze the difference of A2AR expression in tumor size (T1 + T2 vs T3 + T4), lymph node metastasis (N0 vs N1 + N2), HPV infection status (HPV+ vs HPV-), primary HNSCC and recurrence HNSCC (primary vs recurrence), primary HNSCC and TPF chemotherapy specimen (primary vs post TPF), population change of CD4^+^ Foxp3^+^ Tregs and CD4^+^ Foxp3^+^ A2AR^+^ cells from each group, the immunohistochemical staining of Foxp3^+^ and CD8^+^ cells from each group and the increased body weight. The data are presented as the Mean ± SEM, and statistical significance was determined as *P* < 0.05. The correlation of A2AR and HIF-1α, CD73, Foxp3, and CD8 was analyzed by two-tailed Pearson’s statistics. The Kaplan-Meier method and the log-rank test were applied to analyze the overall survival rate (OS) between A2AR high group and A2AR low group. The median of A2AR histoscore was chosen as cut-off. For the hierarchical cluster, the histoscore were converted into −3 to 3 using Microsoft excel. Then, the results were imported and hierarchical analysis was performed with Cluster 3.0. The heatmap was visualized with Java TreeView 1.0.5.

## Results

### A2AR is increased and correlated with clinicopathological parameters in human HNSCC

**Fig. 1:**
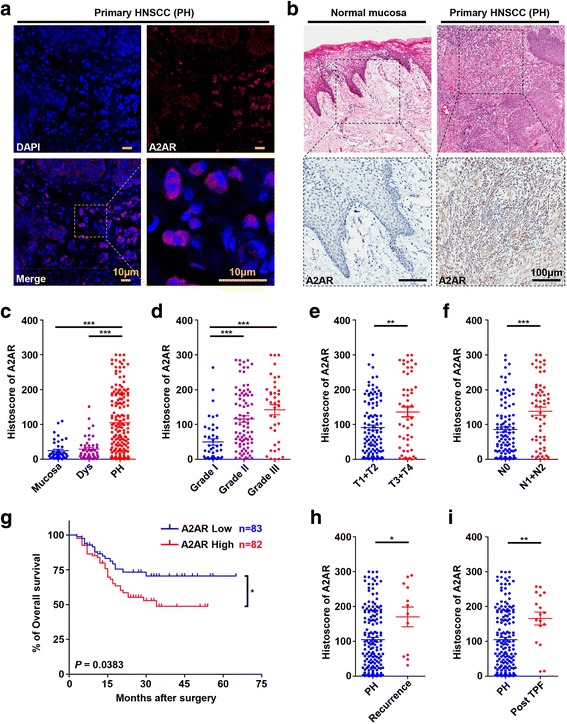
The expression of A2AR is increased and correlated with clinicopathological parameters in human HNSCC. **a** Confocal Immunofluorescence images of A2AR in HNSCC. A2AR expression is located on the tumor infiltrating immune cells (scale bar = 10 μm). **b** Representative HE and immunohistochemical staining of A2AR in human normal oral mucosa and HNSCC tissue (scale bar = 100 μm). **c** Quantification of histoscore of A2AR expression in normal oral mucosa (Mucosa), oral epithelial dysplasia (Dys), and primary HNSCC (PH) tissue. The expression of A2AR was significantly elevated in primary HNSCC tissues as compared with normal oral mucosa or oral epithelial dysplasia (Mean ± SEM, ***, *P* < 0.001, One way ANOVA with post Tukey test). **d-f** The expression of A2AR was correlated with **d** advanced pathological grade, **e** larger tumor size and **f** positive lymph node status in primary HNSCC (Mean ± SEM, **, *P* < 0.01, ***, *P* < 0.001, One way ANOVA with post Tukey test or unpaired *t* test). **g** Kaplan-Meier survival analysis indicated that high expression of A2AR represented unfavorable prognosis of HNSCC patients (*P* = 0.0383). **h** The expression of A2AR was significantly increased in recurrent HNSCC (recurrence, Mean ± SEM, *, *P* < 0.05, unpaired *t* test). **i** The expression of A2AR was significantly increased in HNSCC with induction chemotherapy (post TPF, Mean ± SEM, **, *P* < 0.01 unpaired *t* test). All precise *P* value and the Mean ± SEM was displayed in Table 1

At the first time, we examined the mRNA level of *ADORA2A*, encoding human A2AR, in Estilo’s tongue squamous cell carcinoma (SCC) dataset, a publicly available cancer database [30]. We found that A2AR mRNA level was significantly elevated in tongue SCC as compared with normal oral mucosa (see Additional file 1: Figure S1). Next, the location of A2AR in tumor immune cells was detected by confocal immunofluorescence. A2AR displayed a membrane expression accompanied by a cytoplasmic expression (Fig. 1a). For further analysis, the expression of A2AR in tumor infiltrated immune cells was detected by immunohistochemical (IHC) staining in our HNSCC tissue microarrays (TMAs), which contains 43 normal oral mucosae, 48 dysplasia (Dys) and 165 primary HNSCC (PH) tissues. Digital quantification revealed that the histoscore of A2AR in primary HNSCC was significantly elevated as compared with normal oral mucosa and epithelial dysplasia (Dys, Fig. 1b, c). Remarkably, we noted that increased expression of A2AR was correlated with higher pathological grade (Fig. 1d, Grade I vs. II, *P* < 0.001, Grade I vs. Grade III, *P* < 0.001), larger tumor size (Fig. 1e, T1 + T2 vs T3 + T4, *P* < 0.01), and positive lymph node status (Fig. 1f, N0 vs. N1 + N2, *P* < 0.001). These data clearly indicated that the increased expression of A2AR in HNSCC was correlated with advanced disease stage. To further explore the prognostic value of A2AR in HNSCC, Kaplan-Meier analysis was carried out and the median of A2AR histoscore was selected as a cutoff point. Higher expression of A2AR indicated a rather poorer outcome as compared with lower A2AR expression cohort (*P* = 0.0383, Hazard Ratio: 1.784, 95% CI: 1.038–3.046, Fig. 1g).

HPV-associated HNSCC is a distinct subtype with different intratumoral immune cells infiltration and better prognosis [31]. However, we found no significant correlation between A2AR expression and HPV infection status (see Additional file 2: Figure S2 and Table 1). Meanwhile, no significant association was observed between the histoscore of A2AR in HNSCC and age or gender (Table 1).

**Table 1:** The correlation of A2AR expression with clinicopathologic parameters in HNSCC

Parameters	No.	Mean ± SEM	*P* value
Norma Mucosa	43	23.97 ± 4.106	Mucosa vs. Dys 0.9522
Dysplasia (Dys)	48	28.52 ± 4.661	Mucosa vs. PH < 0.001
Primary HNSCC (PH)	165	104.8 ± 6.823	Dys vs. PH < 0.001
Grade			
I	43	49.84 ± 9.083	I vs. II < 0.001
II	84	115.8 ± 9.275	I vs. III < 0.001
III	38	142.8 ± 15.00	II vs. III 0.2093
Lymph node involvement			
N0	105	85.53 ± 7.646	<0.001
N1 + N2	60	138.6 ± 12.05	
Tumor Size			
T1 + T2	115	91.07 ± 7.109	0.002
T3 + T4	50	136.5 ± 14.65	
Gender			
Male	129	104.7 ± 7.607	0.9610
Female	36	105.5 ± 15.54	
Age			
< 50	44	98.84 ± 14.59	0.5976
≥ 50	121	107.0 ± 7.672	
HPV infection			
Negative	149	102.0 ± 7.011	0.2017
Positive	16	131.5 ± 26.08	
Primary HNSCC (PH)	165	104.8 ± 6.823	(reference)
Recurrent HNSCC (Recurrence)	12	170.2 ± 28.35	0.0144
HNSCC with inductive TPF chemotherapy (post TPF)	17	165.5 ± 18.40	0.0066

Difference among three groups was analyzed by One-way ANOVO followed Tukey test. Difference between two groups was analyzed by un-paired or paired t test

### A2AR is increased in human recurrent HNSCC and HNSCC with induction chemotherapy

Locoregional recurrence and therapy resistance are the major contributors to treatment failures and death of HNSCC patients [32]. Thus, it prompted us to investigate the expression of A2AR in the recurrent HNSCC and HNSCC with induction chemotherapy. 12 cases of recurrent HNSCC tissues, 17 cases of HNSCC tissues with induction chemotherapy were involved in immunohistochemical analysis. Interestingly, as compared with the primary HNSCC tissues, the histoscore of A2AR in recurrent HNSCC tissues (*P* < 0.05) and HNSCC tissues with induction chemotherapy (*P* < 0.01) was significantly increased (Fig. 1h, i). These results indicated that A2AR activation was probably associated with recurrence and chemotherapy resistance.

### A2AR is remarkably correlated with HIF-1α, CD73, CD8 and Foxp3 in primary human HNSCC tissues

**Fig. 2:**
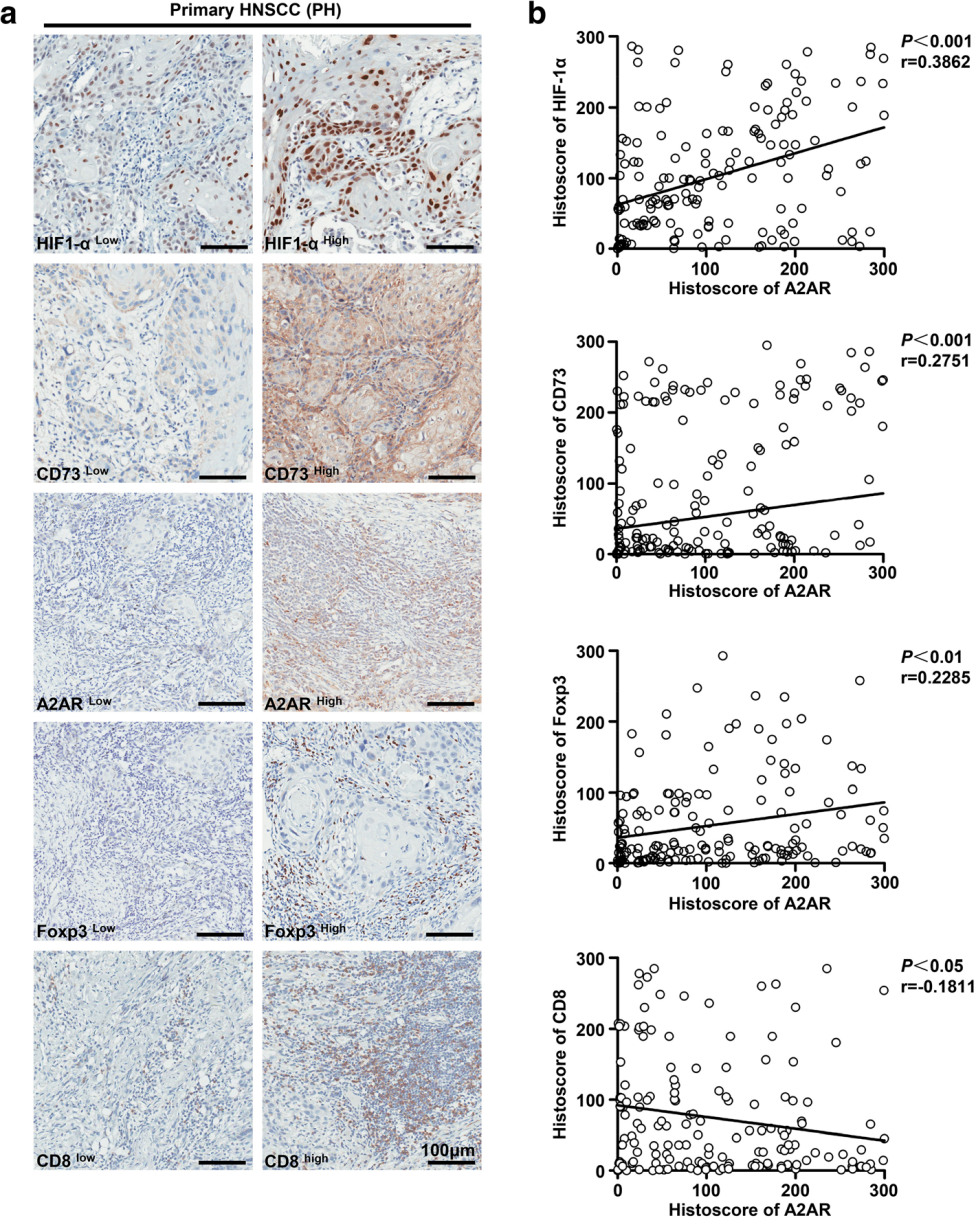
A2AR is remarkably correlated with HIF-1α, CD73, CD8 and Foxp3 in primary human HNSCC tissues. **a** Representative immunohistochemical staining pictures of A2AR, HIF-1α, CD73 and Foxp3 in serial primary HNSCC tissue microarray sections were presented at low or high staining intensities (scale bar = 100 μm). **b** Spearman rank correlation coefficient test and linear tendency test indicated that A2AR was positively correlated with HIF-1α (*P* < 0.001 *r* = 0.3862), CD73 (*P* < 0.001 *r* = 0.2751) and Foxp3 (*P* < 0.01 *r* = 0.2285), but negatively correlated with CD8 (*P* < 0.05 *r* = −0.1811)

It has been reported that hypoxia could induce the expression of HIF-1α and subsequently activated CD73-A2AR adenosine pathway, which benefited to the immune escape of cancer cells [33]. On the basis of this, we examined the association among HIF-1α (hypoxia marker), CD73 (adenosine generator) and A2AR (adenosine receptor) by immunohistochemical analysis in primary HNSCC (PH) serial cutting sections. As shown in Fig. 2a, b, Pearson’s statistical analysis indicated that the expression of A2AR was significantly positively associated with HIF-1α (*P* < 0.001 *r* = 0.3862) and CD73 (*P* < 0.001 *r* = 0.2751). This correlation indicated the synergic activation of the hypoxia-CD73-A2AR pathway in human HNSCC.

Moreover, A2AR expression has been previously reported not only to be associated with the expansion of Tregs, but also to cause CD8^+^ T cells anergy, thus exerting a potent immunosuppressive function [25, 34]. Hence, the relationship among A2AR, CD8 and Foxp3 was evaluated in primary HNSCC serial cutting sections. As expected, the expression of A2AR was significantly correlated with Foxp3 (Fig. 2a, b, *P* < 0.01 *r* = 0.2285), and negatively correlated with CD8 (Fig. 2a, b, *P* < 0.05 *r* = −0.1811). The relationship among these molecules was determined by Hierarchal clustering analysis in primary HNSCC (see Additional file 3: Figure S3). These results indicated that the A2AR activation probably be associated with immunosuppressive status in primary HNSCC.

### Loss of *Tgfbr1* and *Pten* in murine epithelia induces extracellular adenosine pathway activation

**Fig. 3:**
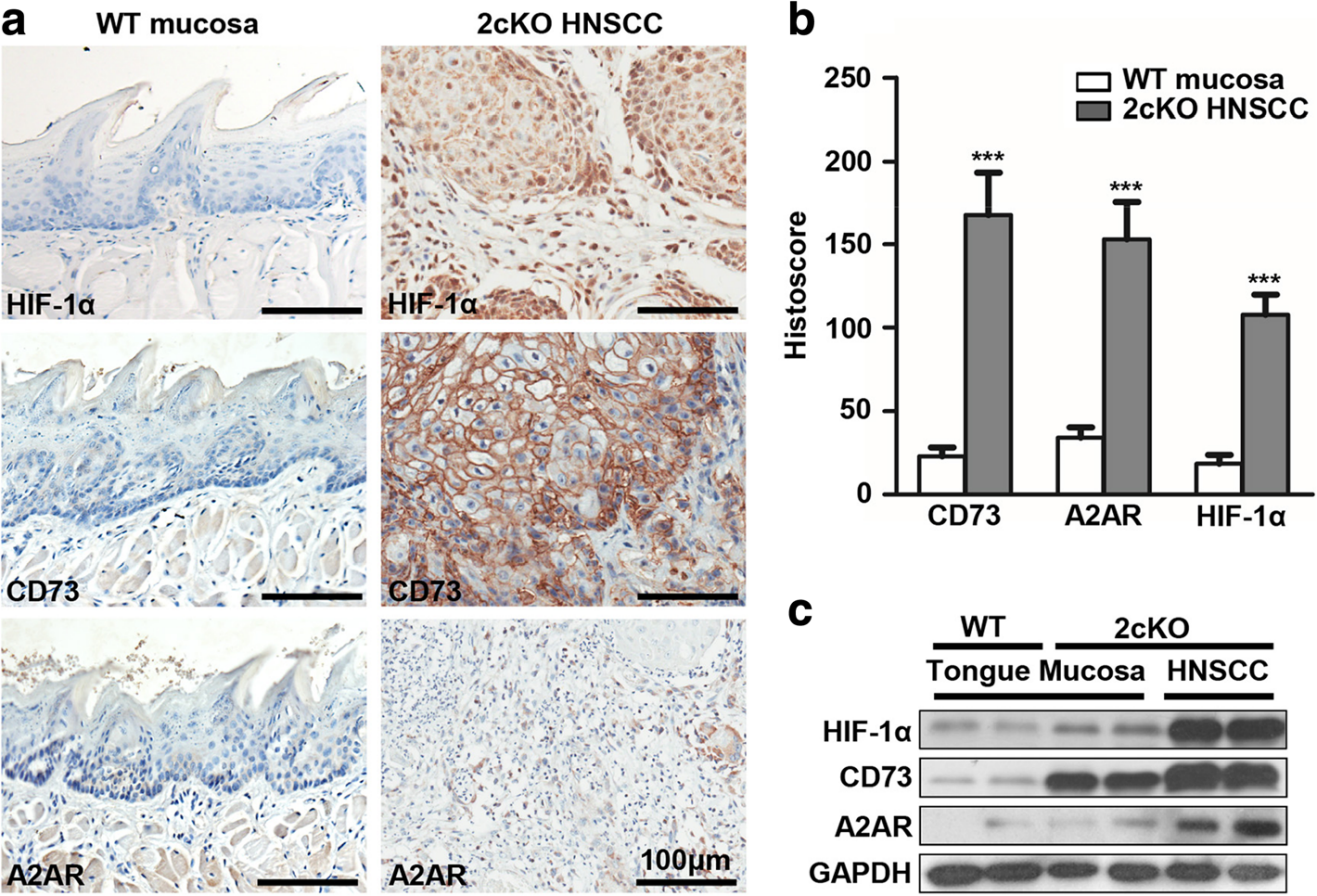
Loss of *Tgfbr1* and *Pten* in murine epithelia induces extracellular adenosine pathway activation. **a** Representative pictures of HIF-1α, CD73 and A2AR immunohistochemical staining of normal tongue mucosa from wild type (WT) mice and HNSCC from *Tgfbr1* and *Pten* double conditional knockout (2cKO) tumor bearing mice (each group *n* = 6, left, scale bar = 100 μm). **b** The digital quantification of the histoscore of HIF-1α, CD73 and A2AR. The expression of HIF-1α, CD73 and A2AR was significantly elevated in the tumor from 2cKO mice as compared with the normal tongue mucosa from the WT mice (Mean ± SEM, ***, *P* < 0.001, unpaired *t* test). **c** Western blot indicated that combined deletion of *Tgfbr1* and *Pten* in head and neck epithelial obviously elevated the protein level of HIF-1α, CD73 and A2AR. GAPDH was used as loading control

Loss of *TGFBR1* and *PTEN* is a common event in human HNSCC [35]. On the basis of this, we generated the immunocompetent combined *Tgfbr1/Pten* conditional knockout (2cKO) mouse model by crossing *K14-CreER* ^tam^; *Tgfbr1* ^flox/flox^ (*Tgfbr1* cKO) mice with *Pten* ^flox/flox^ mice, which spontaneously develops HNSCC with full penetration, and is suitable for preclinical intervention, especially for cancer immunotherapy research [29]. In order to assess the expression of HIF-1α, CD73 and A2AR in 2cKO mouse model, we detected the protein expression of CD73 and A2AR by IHC staining and western blot. HIF-1α and CD73 were obviously elevated in the tumor cells of 2cKO mice as compared with the normal tongue mucosa from the wild type mice, and A2AR was up-regulated in the infiltrating immune cells (Fig. 3a with quantification in Fig 3b). Additionally, the western blot indicated that the protein level of HIF-1α, CD73 and A2AR was upregulated in 2cKO tumor bearing mice as compared with the wild type mice (Fig. 3c). These results provided an evidence that hypoxia-CD73-A2AR pathway was activated in the HNSCC mouse model.

### The expression of A2AR is upregulated on CD4^+^ Foxp3^+^ Tregs and CD8^+^ T cells in *Tgfbr1*/*Pten* 2cKO tumor bearing mice

**Fig. 4:**
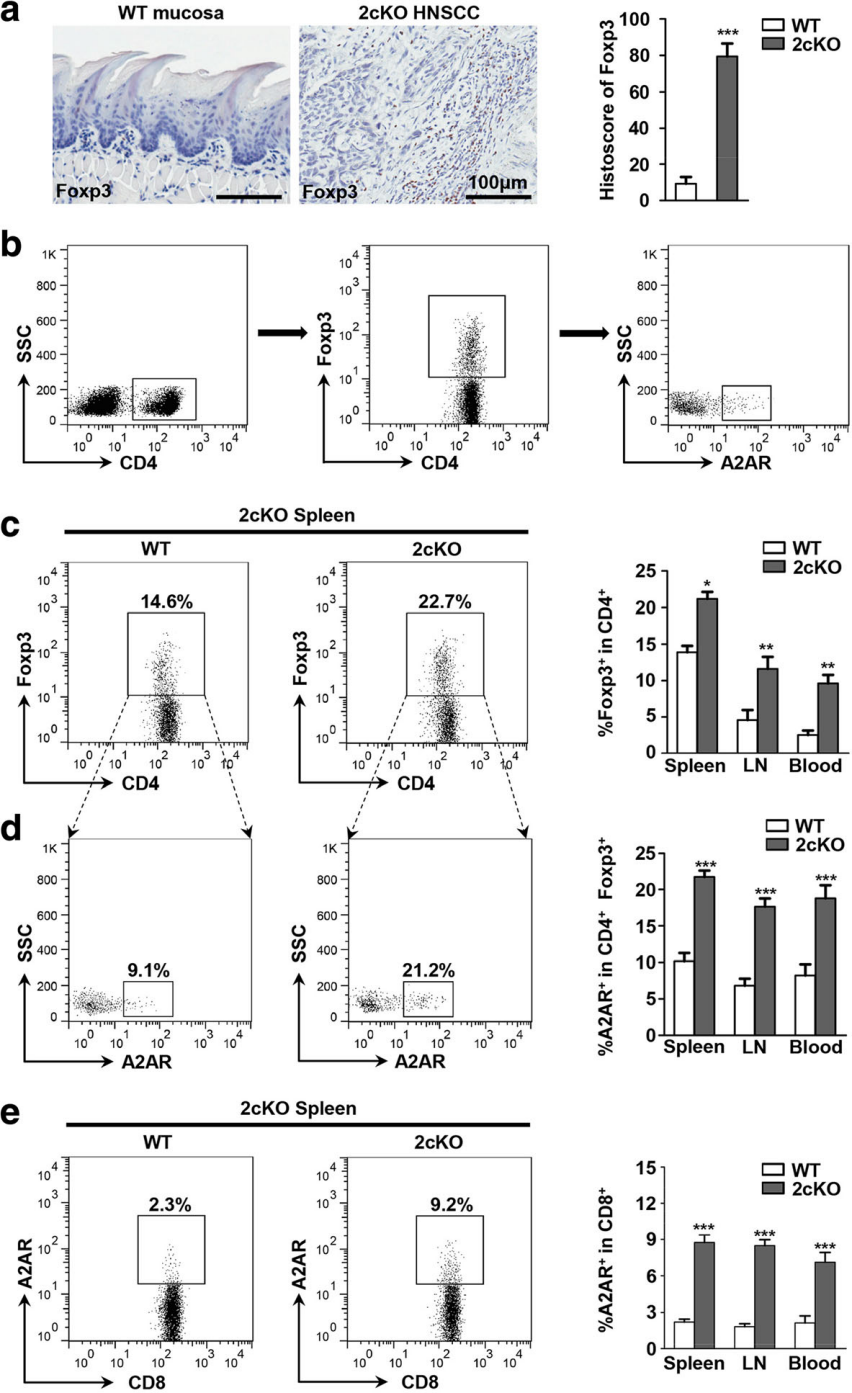
A2AR is upregulated on Tregs and CD8^+^ T cells in 2cKO tumor bearing mice. **a** Representative image of Foxp3 immunohistochemical staining with quantification in normal tongue mucosa from WT mice and HNSCC from 2cKO tumor bearing mice (each group *n* = 6, scale bar = 100 μm, Mean ± SEM, ***, *P* < 0.001, unpaired *t* test). **b** The gating strategy of A2AR expression on CD4^+^ Foxp3^+^ Tregs. **c** Representative dot plots of CD4^+^ Foxp3^+^ Tregs in spleen of WT mice and 2cKO tumor bearing mice (left). Quantitative analysis of the CD4^+^ Foxp3^+^ Tregs population in spleen, lymph nodes, and peripheral blood of 2cKO tumor bearing mice as compared with WT mice (right, each group *n* = 6, Mean ± SEM, *, *P* < 0.05, **, *P* < 0.01, unpaired *t* test). **d** Representative dot plots of A2AR expression on CD4^+^ Foxp3^+^ Tregs in WT mice and 2cKO tumor bearing mice (left). Quantitative analysis of the frequency of A2AR expression on CD4^+^ Foxp3^+^ Tregs in spleen, lymph node, and peripheral blood of WT mice and 2cKO tumor bearing mice (right, each group *n* = 6, Mean ± SEM, *** *P* < 0.001, unpaired *t* test). **e** Representative dot plots of A2AR expression on CD8^+^ T cells in WT mice and 2cKO tumor bearing mice (left). Quantitative analysis of the frequency of A2AR expression on CD8^+^ T cells in spleen, lymph node, and peripheral blood of WT mice and 2cKO tumor bearing mice (right, each group *n* = 6, Mean ± SEM, *** *P* < 0.001, unpaired *t* test)

Paracrine effects of TGF-β signaling are believed to play a pivotal role in cancer promoting effects via stimulation of inflammation and escape from immunosurveillance [36]. Conditional knockout of *Tgfbr1* in epithelia induces an enhanced paracrine effect of TGF-β1 on tumor stroma [37]. Considering the role of immunosuppressive TGF-β signaling in Tregs induction, we assessed the expression of Foxp3 in the tumor site of 2cKO tumor bearing mice. As compared with the normal mucosa from the wild type mice, the expression of Foxp3 was obviously increased (Fig. 4a). To further evaluate the systemic immunosuppressive status of the 2cKO tumor bearing mice, we calculated the population of CD4^+^ Foxp3^+^ Tregs in spleen, lymph nodes and peripheral blood from wild type mice or 2cKO tumor bearing mice respectively by quantitative flow cytometric analysis. As compared with the wild type, significant increase of CD4^+^ Foxp3^+^ Tregs population was found in the spleen, lymph nodes and peripheral blood of 2cKO tumor bearing mice (Fig. 4b, c). Given the close relationship between A2AR and Foxp3 in human HNSCC sample, the expression of A2AR on the surface of CD4^+^ Foxp3^+^ Tregs was subsequently assessed. The number of Tregs expressing A2AR was increased in the 2cKO tumor bearing mice as compared with wild type mice (Fig. 4d).

cAMP-elevated signaling through A2AR results in inhibition of TCR-triggered activation of T cells and of many effector functions, such as proliferation, expansion and antitumor cytokine secretion [34]. Additionally, our previous study revealed that the population of CD8^+^ T cells was significantly decreased during the tumorigenesis and tumor progression in the 2cKO mice [29, 38]. This prompted us to investigate the expression of A2AR in the CD8^+^ T cells in the 2cKO tumor bearing mice. As compared with WT mice, the frequency of A2AR was significantly elevated on the surface of CD8^+^ T cells in the spleen, lymph nodes and peripheral blood of 2cKO bearing mice (Fig. 4e). This result indicated that A2AR might exert the immunomodulatory function by influencing CD8^+^ T cells in 2cKO tumor bearing mice.

### Pharmacological blockade of A2AR delays tumor growth in HNSCC mouse model

**Fig. 5:**
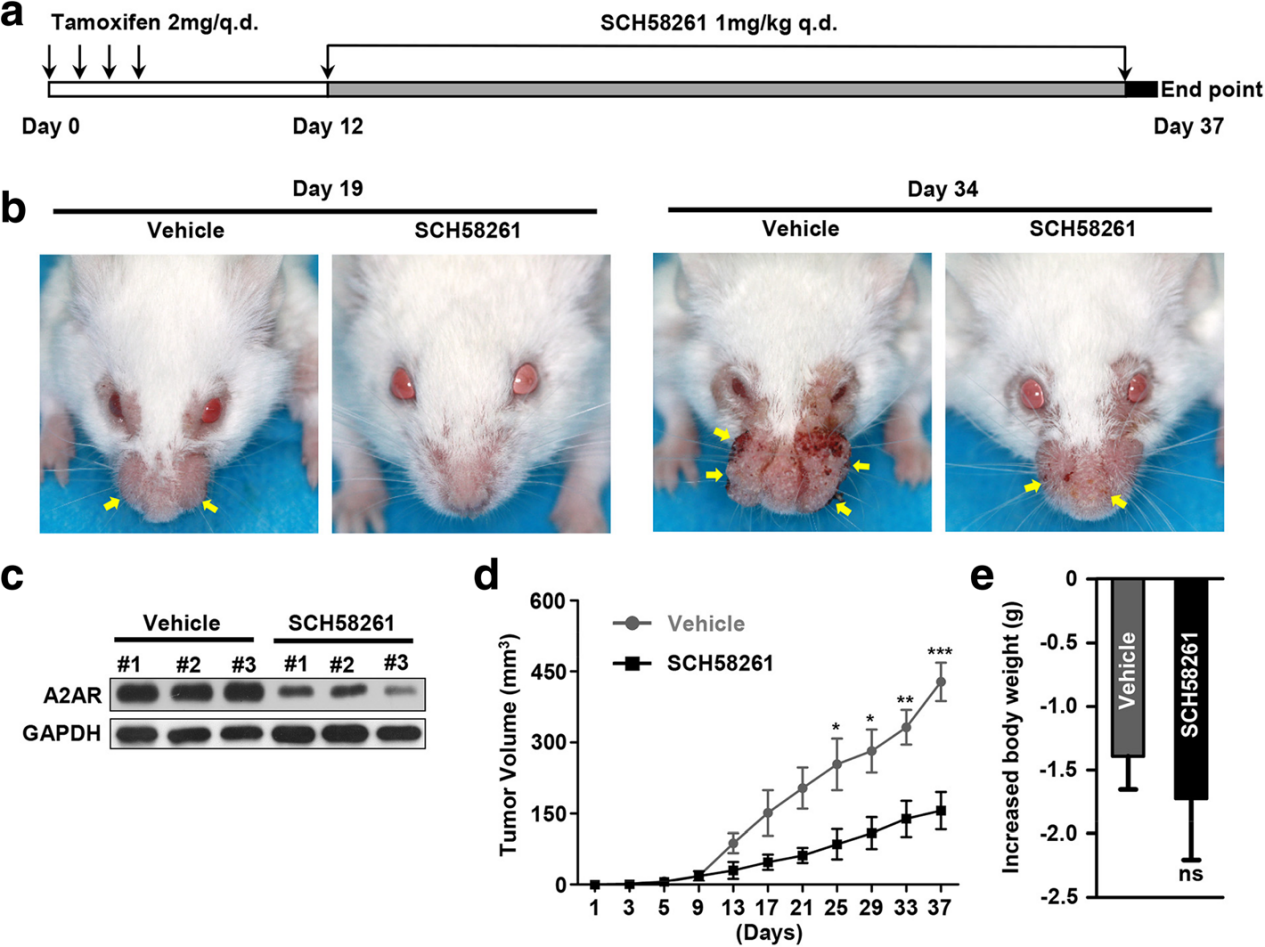
Pharmacological blockade of A2AR delays tumor growth in HNSCC mouse model. **a** Schematic representation of tamoxifen oral gavage and SCH58261 treatment of the spontaneous HNSCC mouse model. **b** Representative photos of tumor (yellow arrowhead) in mice treated with DMSO (Vehicle, *left*) or SCH58261 (*right*) at day 19 and day 34 after tamoxifen oral gavage. **c** The A2AR inhibition efficiency was tested by western blotting (GAPDH was used as loading control). SCH58261 treatment obviously decreased the protein level of A2AR in the lysis of the tumor. **d** Tumor volumes over time of vehicle group and SCH58261 treated group (*n* = 6, each group). The statistical significance reached since day 25(Mean ± SEM, * *P* < 0.05, **, *P* < 0.01, ***, *P* < 0.001). **e** Drug toxicity was presented as increased body weight of the mice in vehicle group and SCH58261 treated group (ns = no significance, unpaired *t* test)

A2AR blockade was reported as a promising strategy for cancer immunotherapy in some studies [15, 18–20]. To further investigate the chemopreventive efficacy of A2AR blockade in vivo, immunocompetent *Tgfbr1/Pten* 2cKO HNSCC mice were employed. As is schematically presented in Fig. 5a, tamoxifen induction was applied for tumorigenesis via oral gavage in 5 consecutive days. A week later, we initiated treatment with 1 mg/kg A2AR antagonist SCH58261 (treatment group) or DMSO (vehicle group) by intraperitoneal injection every day for 25 days (each group *n* = 6 mice). The tumor growth was mainly restricted to the head and neck area of mice (Fig. 5b, yellow arrowhead). Tumor growth was assessed every other day after tamoxifen gavage. All mice were euthanized for study at the endpoint (Day37). The protein inhibition efficacy of A2AR antagonist was verified by western blot (Fig. 5c). Based on the measurement of tumor volume, the tumor growth of the SCH58261 group was significantly delayed after day 25 as compared with the vehicle group (Fig. 5d). The increased body weight of vehicle group and SCH58261 treated group was assessed, and the data showed that SCH58261 caused no extra toxic effect (Fig. 5e). Taken together, these preclinical data suggested that A2AR blockade might be a potential approach for treating HNSCC.

### A2AR blockade reduces CD4^+^ Foxp3^+^ Tregs in HNSCC mouse model

**Fig. 6:**
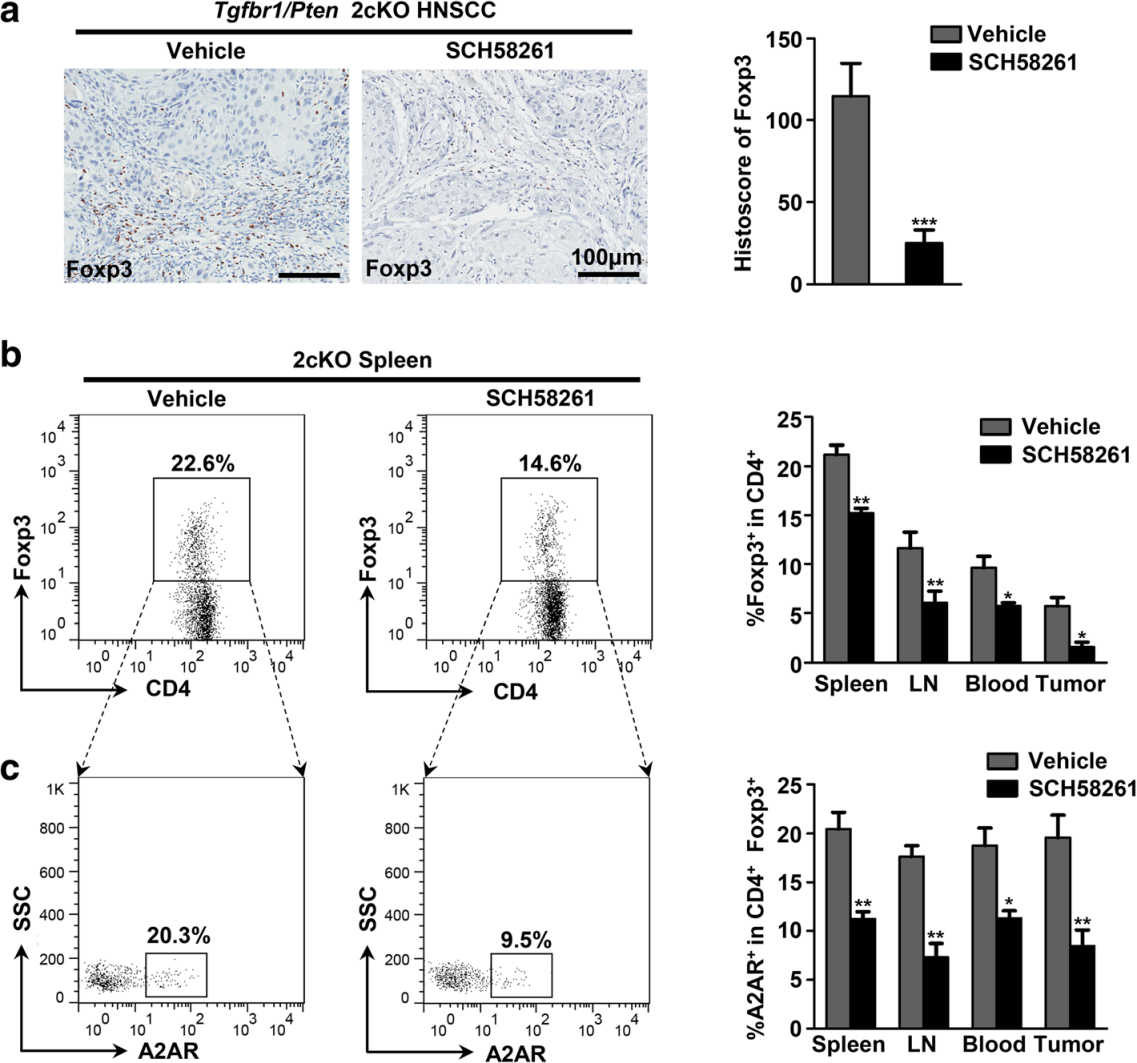
A2AR blockade decreases CD4^+^ Foxp3^+^ Tregs in HNSCC mouse model. **a** Representative photos of Foxp3 immunohistochemical staining of vehicle group and SCH58261 treated group (left). The histoscore quantification of Foxp3 was showed by bar graph (*right*, each group *n* = 6, Mean ± SEM, ***, *P* < 0.001, unpaired *t* test). **b** Representative dot plots of CD4^+^ Foxp3^+^ Tregs in spleen of Vehicle group and SCH58261 group (*left*). Bar graphs showed quantitative analysis of the percentage of CD4^+^ Foxp3^+^ Tregs in spleen, lymph node, peripheral blood and tumor from each group (right, each group *n* = 6, Mean ± SEM, *, *P* < 0.05, **, *P* < 0.01, unpaired *t* test). **c** Representative dot plots of A2AR on CD4^+^ Foxp3^+^ Tregs in spleen of mice from vehicle group and SCH58261 group (*left*). Quantification of the frequency of A2AR on CD4^+^ Foxp3^+^ Tregs in each group is presented by bar graph (right, each group *n* = 6, Mean ± SEM, *, *P* < 0.05, **, *P* < 0.01, unpaired *t* test)

It has been reported that A2AR stimulation by agonist numerically and functionally enhanced the immunosuppressive mechanism mediated by Tregs [25]. Based on this finding, we quantified the histoscore of Foxp3 cells in the cancerous area from the vehicle mice and SCH58261 treated mice respectively by immunohistochemical staining. SCH58261 treatment significantly reduced the expression of Foxp3, indicating a decrease of Tregs accumulation (Fig. 6a). Subsequently, the accurate population of CD4^+^ Foxp3^+^ Tregs in vehicle mice and SCH58261 treated mice were analyzed by flow cytometry. As compared with vehicle group, blockade of A2AR by SCH58261 significantly reduced the population of CD4^+^ Foxp3^+^ Tregs in spleen, lymph nodes, peripheral blood and tumors (Fig. 6b). Additionally, flow cytometric analysis detected that the expression of A2AR was dramatically decreased on the surface of CD4^+^ Foxp3^+^ Tregs in SCH58261 treated 2cKO mice (Fig. 6c). These results demonstrated that A2AR blockade is an effective approach for reducing Tregs in 2cKO tumor bearing mice. Except for Tregs, tumor-associated macrophages (TAMs) and myeloid-derived suppressor cells (MDSCs) were recruited in the tumor microenvironment and facilitated tumor-mediated immune escape in HNSCC [39, 40]. To explore whether the blockade of A2AR influenced the population of TAMs and MDSCs, we analyzed their frequencies in 2cKO tumor bearing mice. Indeed, the percentages of TAMs (CD11b^+^ F4/80^+^) and MDSCs (CD11b^+^ Gr-1^+^) were significantly increased in vehicle group as compared with wild type mice. However, SCH58261 treatment could not significantly reduce the frequency of TAMs and MDSCs (see Additional file 4: Figure S4).

### A2AR blockade enhances the anti-tumor response of CD8^+^ T cells in HNSCC mouse model

**Fig. 7:**
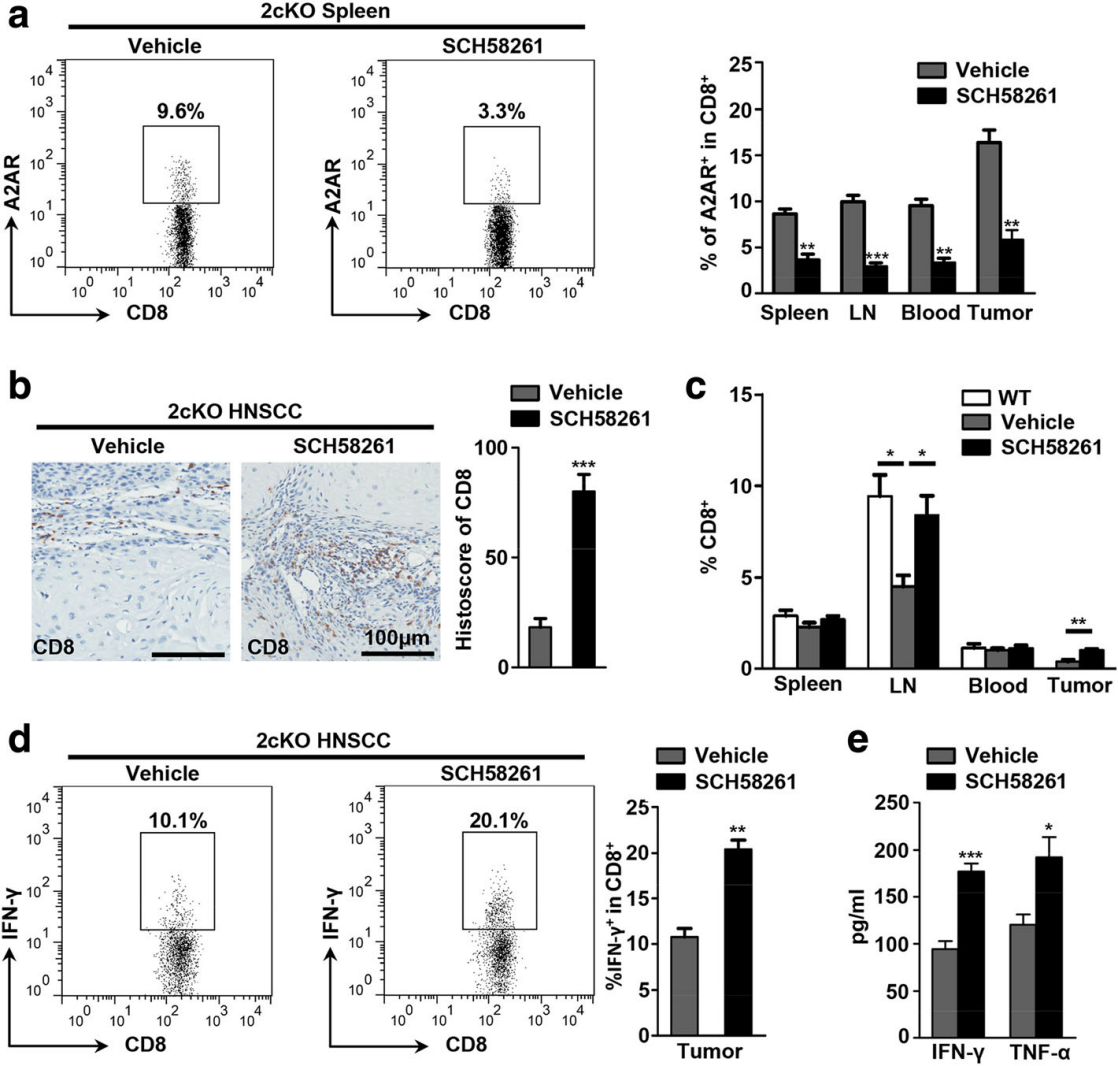
A2AR blockade enhances the anti-tumor response of CD8^+^ T cells in HNSCC mouse model. **a** Dot plots of A2AR^+^ CD8^+^ T cells in spleen from vehicle group and SCH58261 group (left). Quantification of the percentage of A2AR^+^CD8^+^ T cells in spleen, lymph node, peripheral blood and tumor was displayed with bar graph (right, each group *n* = 6, Mean ± SEM, **, *P* < 0.01, ***, *P* < 0.001, unpaired *t* test). **b** Representative photos of CD8 immunohistochemical staining in the tumor from vehicle group or SCH58261 group were shown (scale bar = 100 μm)). The histoscore of CD8 of each group were calculated and presented with bar graph (each group *n* = 6, Mean ± SEM, ***, *P* < 0.001, *t* test). **c** Quantifications of the population of CD8^+^ T cells in spleen, lymph node, peripheral blood and tumor from WT mice, Vehicle group and SCH58261 group (each group *n* = 6, Mean ± SEM, *, *P* < 0.05, **, *P* < 0.01, unpaired *t* test). **d** Dot plots of IFN-γ^+^ CD8^+^ T cells in tumor infiltrating lymphocytes (TILs) from vehicle group and SCH58261 group (left). Quantification of the percentage of IFN-γ^+^ CD8^+^ T was displayed with bar graph (right, each group *n* = 6, Mean ± SEM, **, *P* < 0.01, unpaired *t* test). **e** Production of IFN-γ and TNF-α of the CD8^+^ T cells from SCH58261 treated 2cKO tumor bearing mice was significantly increased as compared with the mice from vehicle group (each group *n* = 6, Mean ± SEM, *, *P* < 0.05, **, *P* < 0.01, unpaired *t* test)

Essentially, extracellular adenosine disables the cytotoxic effector functions of CD8^+^ T cells predominately through A2AR signaling, contributing to the immune evasion and escape of tumor cell [40]. On the basis of this, we evaluated the A2AR expression on the surface of CD8^+^ T cells in the tumor bearing mice treated with vehicle or SCH58261 respectively. A2AR expression on the surface of CD8^+^ T cells was significantly reduced in the spleen, lymph nodes, peripheral blood and tumor site of SCH58261 treated 2cKO tumor bearing mice as compared with the vehicle group (Fig. 7a). Additionally, our results indicated that the accumulation of CD8^+^ T cells was remarkably increased in the lymph nodes and the tumor site from the SCH58261 treated 2cKO mice (Fig. 7b, c). Moreover, we evaluated the population of CD8^+^ IFN-γ^+^ T cells in vehicle group and SCH58261 treated group. Data showed that the population of CD8^+^ IFN-γ + T cells was significantly increased after SCH58261 treatment (Fig 7d). By immunomagnetic sorting, we isolated the tumor infiltrated CD8^+^ T cells from the vehicle group or the SCH58261 treated group. The production levels of IFN-γ and TNF-α were measured by enzyme-linked immunosorbent assay (ELISA). We found that SCH58261 treatment significantly increased the production of IFN-γ and TNF-α (Fig 7e). Taken together, these data revealed that A2AR blockade numerically and functionally enhanced the CD8^+^ T cells in murine HNSCC.

## Discussion

Adenosine plays crucial roles in the establishment of an immunosuppressive tumor microenvironment, benefiting the progression of cancer [41]. Tissue hypoxia seems to be essential to the increase of intratumoral adenosine levels [24]. On one hand, hypoxia elevates the expression of adenosine generation pathway CD39 and CD73 [33, 42]. On the other hand, hypoxia decreases adenosine kinase and inhibits the conversion of adenosine [43]. As a consequence, specific adenosine receptors such as A2AR are activated by increased levels of extracellular adenosine in tumor microenvironment [43]. In the present study, elevated expression of A2AR, which was positively correlated with HIF-1α and CD73, was detected in HNSCC tissue microarrays, indicating the activation of hypoxia-CD73-A2AR pathway. Hypoxia appears quite frequently in a variety of solid tumors when tumor growth exceeds the angiogenic growth [44, 45]. An earlier study demonstrated that nuclear overexpression of HIF-1α was detected in 69.64% of analyzed oral squamous cell carcinoma (OSCC), being positively correlated with the rate of tumor progression (tumor size, lymph node metastasis and histological differentiation) [46]. In the current study, overexpression of A2AR was linked to larger tumor size, lymph node metastasis and pathological grade. Considering the positive correlation between A2AR and HIF-1α in HNSCC tissues, we suggested that A2AR interfere the tumor progression rate partially depend on hypoxia status. To date, there is no convincing research indicating that A2AR overexpression was correlated with poor clinical outcome in HNSCC. In the present study, Kaplan-Meier analysis data implicated high expression of A2AR was associated with unfavorable clinical prognosis.

Under the influence of adenosine pathway, CD8^+^ T cells become less cytotoxic with decreased TCR signaling and production of proinflammatory cytokines, such as IFN-γ [47]. Considering the immunosuppressive role of A2AR in cancerous tissues, we subsequently assessed the correlation between A2AR and CD8 and found that A2AR expression was negatively correlated with the CD8 expression in human HNSCC tissues. To some extent, these results suggested A2AR might play an immunosuppressive role through influencing CD8^+^ T cells population in HNSCC. Notably, it has been earlier reported that hypoxia-A2AR pathway was not only an immunosuppressive signaling that inhibits the TCR signaling, but also facilitated the development of immunosuppressive Tregs [48]. Moreover, a recent study demonstrated that in vitro A2AR stimulation by agonist numerically and functionally enhanced the Tregs sorted from the human peripheral blood [25]. These studies emphasized the regulatory role on Tregs expansion and function by A2AR signaling. Indeed, in this present study, the positive correlation between the expression of A2AR and Foxp3 indicated the potential relevance between A2AR signaling and Tregs in HNSCC.

Alterations of PTEN/PI3K/AKT/mTOR pathway and TGF-β1 are the most frequent molecular events during HNSCC tumorigenesis and progression [49]. In the previous study, by combining knockout of *Tgfbr1* and *Pten* (2cKO), we constructed a spontaneous immune competent HNSCC mouse model, which was suggested as an appropriate pre-clinical animal model for HNSCC research. The emerging tumors were not only pathologically indistinguishable from the human HNSCC, but also presented major molecular alternations in human HNSCC [35]. In the present study, deletion of *Tgfbr1* and *Pten* in mouse head and neck epithelia activated hypoxia-CD73 pathway and consequently induced the elevation of A2AR on the immune cells, including CD4^+^ Foxp3^+^ Tregs and CD8^+^ T cells. Additionally, deletion of *Tgfbr1* in murine head and neck epithelia resulted in enhanced paracrine effect of TGF-β1 in tumor stroma, which facilitated the immunosuppressive status and promoted the tumor progression [37]. Given that the development of Tregs is under the influence of various inductive signals, most importantly TGF-β1 [50], we found a significantly increased population of CD4^+^ Foxp3^+^ Tregs in the tumor of 2cKO mice. It has been reported that A2AR stimulation enhanced the proliferation of Tregs [25]. In the present study, elevated A2AR was detected on the surface of CD4^+^ Foxp3^+^ Tregs in 2cKO tumor bearing mice, emphasizing the potential role of A2AR signaling in regulating the expansion or functions of Tregs in HNSCC. These findings also provided us with the rationale for decreasing Tregs by A2AR antagonist. Indeed, pharmacological blockade of A2AR by antagonist repressed the tumor growth of 2cKO mice and reduced the population of CD4^+^ Foxp3^+^ Tregs. Meanwhile, an enhanced anti-tumor response of CD8^+^ T cells was observed in 2cKO tumor bearing mice treated with A2AR antagonist SCH58261, indicating the improvement of the immunosuppressive status. This result was partially in accordance with the study that A2AR protected tumor cells from anti-tumor CD8^+^ T cells [15]. It has been reported that selective deletion of A2AR on myeloid cells caused potent tumor rejection which was associated with significant increases of MHC II and IL-12 expression in tumor-associated macrophages (TAMs) and reductions in IL-10 expression in TAMs, dendritic cells (DCs) and myeloid-derived suppressor cells (MDSCs) [51]. In the current study, although the populations of MDSCs and TAMs were significantly increased in 2cKO tumor bearing mice, the A2AR antagonist was unable to decrease the population of these cells. These results indicated that A2AR blockade probably did not affect the expansion of immunosuppressive myeloid cells in HNSCC. However, the effect of A2AR signaling on the functions of myeloid cells needed additional studies.

Cytotoxic chemotherapeutic agents are widely employed in the war for fighting against cancer [52]. Nevertheless, emerging evidence has indicated cytotoxic agents altered the local immune state, interfering the response of treatment [53]. Several cytotoxic chemotherapeutic agents, including 5-FU, appear to produce an in situ vaccination as a consequence of their initial cytotoxic effect and to facilitate an immunogenic cell death (ICD) [52]. During this process, the release of ATP has been identified as a critical mediator [54]. ATP was eventually catabolized to immunosuppressive adenosine by CD39 and CD73 pathway, which are frequently activated by hypoxia in tumor microenvironment, and subsequently changing the immune status in tumor microenvironment. Of interest, we detected a significant up-regulation of A2AR in the HNSCC tissues with induction chemotherapy, indicating that A2AR may facilitate drug resistance probably by altering the immune status in tumor microenvironment. This phenomenon may reflect the potential therapeutic value of combining use of A2AR antagonist and conventional chemotherapeutic reagents in the treatment for HNSCC. In addition, inhibition of immune checkpoints still leaves T cells vulnerable to multi-faceted and powerful immunosuppression by hypoxia-adenosine pathway [55]. Inhibitor of hypoxia-A2-adenosinergic pathway may decrease the intensity of other immunosuppressive factors including CTLA-4 or TGF-β1. This hypothesis was supported by recent studies indicating that CTLA-4 or PD-1 blockade combined with the inhibition of the extracellular adenosine or A2AR/A2BR signaling resulted in a stronger anti-tumor effect [56, 57]. A phase I clinical trial (NCT02655822) of A2AR antagonist (PBF-509 and CPI-444) alone or with immune checkpoint inhibitor (atezolizumab, a PD-L1 inhibitor) is currently recruiting participants to study the clinical efficiency of A2AR blockade for solid tumor including HNSCC.

## Conclusion

In summary, our study revealed the elevated expression of A2AR in human HNSCC tissues, which was correlated with the advanced pathological grade, larger tumor size, positive lymph node status and poor prognosis of HNSCC patients. Moreover, deletion of *Tgfbr1* and *Pten* in murine epithelia activated HIF-1α, CD73 and subsequently induced A2AR overexpression in tumor infiltrating immune cells, accumulating immunosuppressive CD4^+^ Foxp3^+^ Tregs in the stroma of tumors. Furthermore, pharmacological blockade of A2AR in vivo by antagonist SCH58261 repressed the tumor growth, inducing a reduction of CD4^+^ Foxp3^+^ Tregs and an enhanced anti-tumor response of CD8^+^ T cells. Hence, our results provided preclinical evidence that A2AR probably be a potential immunotherapeutic target for treatment of HNSCC.

## Additional files

Additional file 1: Fig. S1. *ADORA2A* mRNA level in human tongue squamous cell carcinoma. *ADORA2A* mRNA level in Estilo’s tongue squamous cell carcinoma (SCC) dataset (*P* = 0.001) (JPEG 200 kb)

Additional file 2: Fig. S2. The relationship of A2AR expression and HPV status. **a** Represent immunohistochemistry image of p16 and A2AR in HPV positive (HPV+) and HPV negative (HPV-) sample. **b** The expression of A2AR was not related to HPV infection status (HPV- vs. HPV+, Mean ± SEM, ns = no significance, unpaired *t* test) (JPEG 690 kb)

Additional file 3: Fig. S3. Hierarchical clustering of HIF-1α, CD73, A2AR, CD8 and Foxp3 histoscore in primary HNSCC. The relationship among HIF-1α, CD73, A2AR, CD8 and Foxp3 was determined by Hierarchal clustering analysis in primary HNSCC (*n* = 165) (JPEG 404 kb)

Additional file 4: Fig. S4. A2AR blockade is unable to influence the population of MDSCs and TAMs. **a** Quantification of MDSCs (CD11b^+^ Gr1^+^) in spleen, lymph node (LN), peripheral blood and tumor from wild type mice (WT) and 2cKO tumor bearing mice treated with DMSO (vehicle group) or with SCH58261(each group *n* = 6, Mean ± SEM, *, *P* < 0.05, ***, *P* < 0.001, ns = no significance, one way ANOVA with post Tukey test). **b** Quantification of TAMs (CD11b^+^ F4/80^+^) in spleen, lymph node (LN), peripheral blood and tumor from WT mice and 2cKO tumor bearing mice treated with DMSO or with SCH58261 (Mean ± SEM, *, *P* < 0.05, ***, *P* < 0.001, ns = no significance, one way ANOVA post Tukey test) (JPEG 288 kb)

## Abbreviations

A2AR: Adenosine A2A receptor
A2BR: Adenosine A2B receptor
HNSCC: Head and neck squamous cell carcinoma
HPV: Human papillomavirus
MDSCs: Myeloid-derived suppressor cells
OSCC: Oral squamous cell carcinoma
TAMs: Tumor associated macrophages
TILs: Tumor infiltrating lymphocytes
Tregs: Regulatory T cells
