# Supplementary figures and images for: Blockade of adenosine A2A receptor enhances CD8+ T cells response and decreases regulatory T cells in head and neck squamous cell carcinoma
Source: Mol Cancer. 2017 Jun 7;16:99. doi: 10.1186/s12943-017-0665-0 (PMC5461710; doi:10.1186/s12943-017-0665-0)

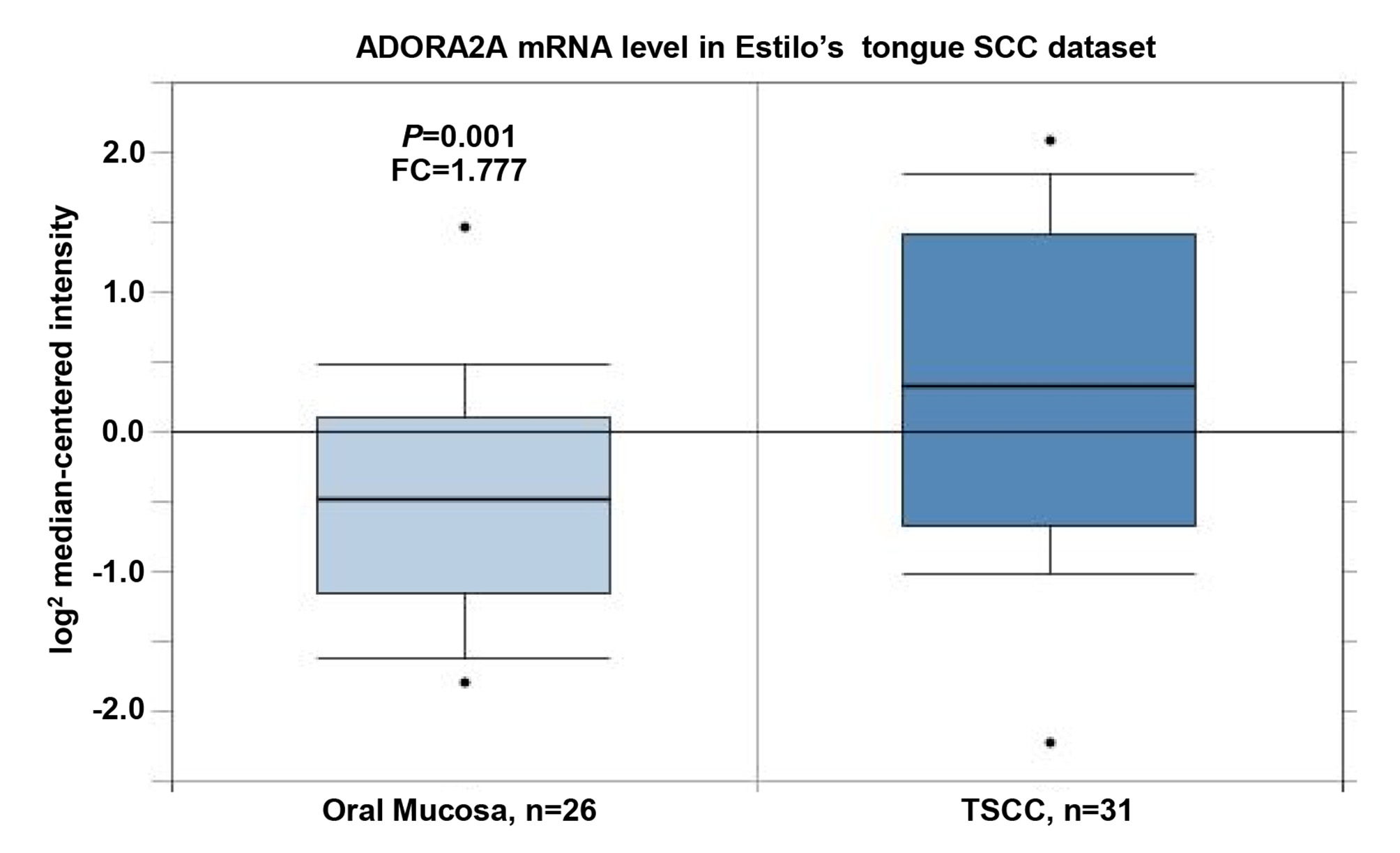

Supplement: Supplementary file 1 — ADORA2A mRNA level in human tongue squamous cell carcinoma. ADORA2A mRNA level in Estilo’s tongue squamous cell carcinoma (SCC) dataset (P = 0.001) (JPEG 200 kb) [file 12943_2017_665_MOESM1_ESM.jpg]

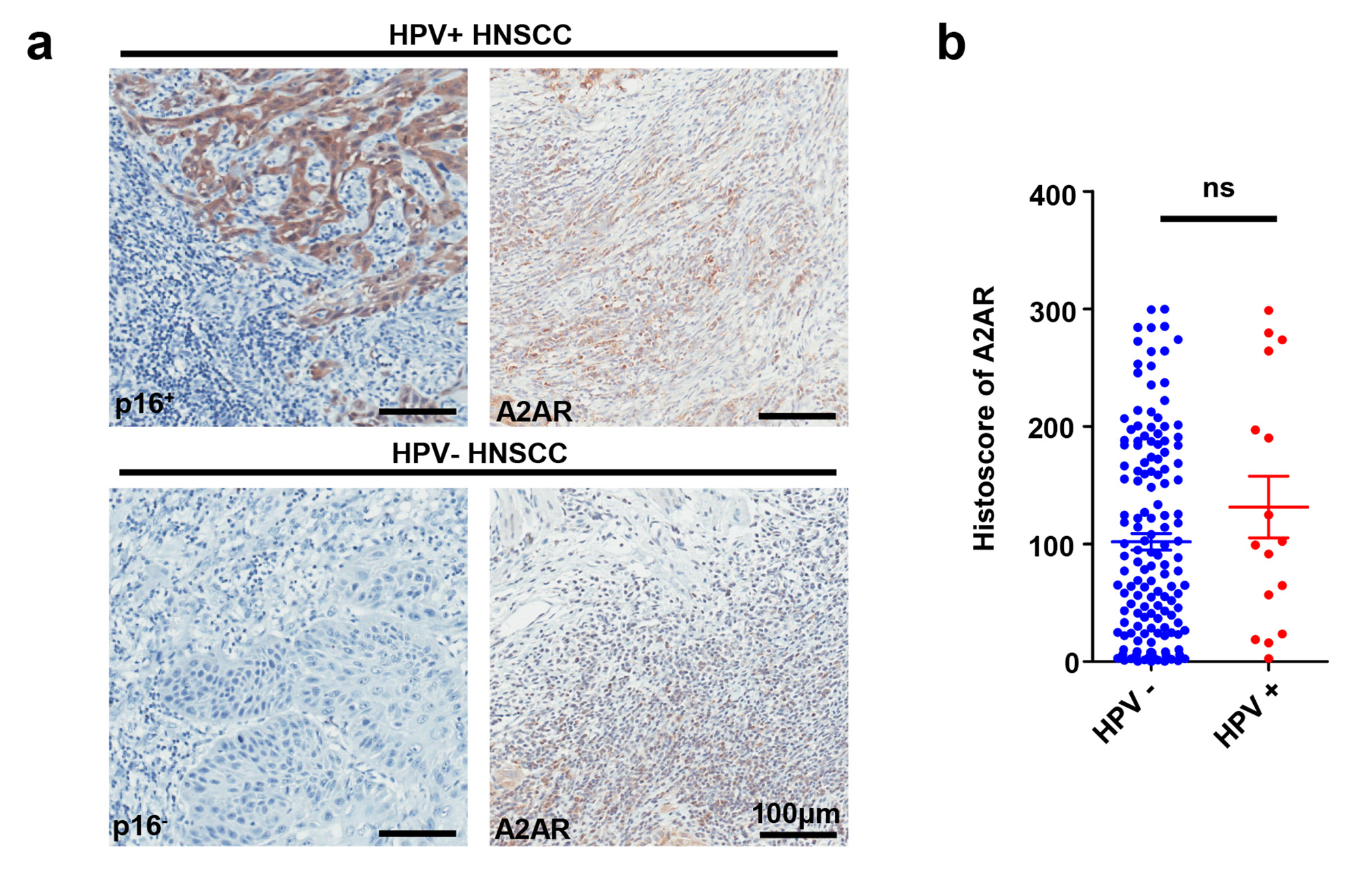

Supplement: Supplementary file 2 — The relationship of A2AR expression and HPV status. a Represent immunohistochemistry image of p16 and A2AR in HPV positive (HPV+) and HPV negative (HPV-) sample. b The expression of A2AR was not related to HPV infection status (HPV- vs. HPV+, Mean ± SEM, ns = no significance, unpaired t test) (JPEG 690 kb) [file 12943_2017_665_MOESM2_ESM.jpg]

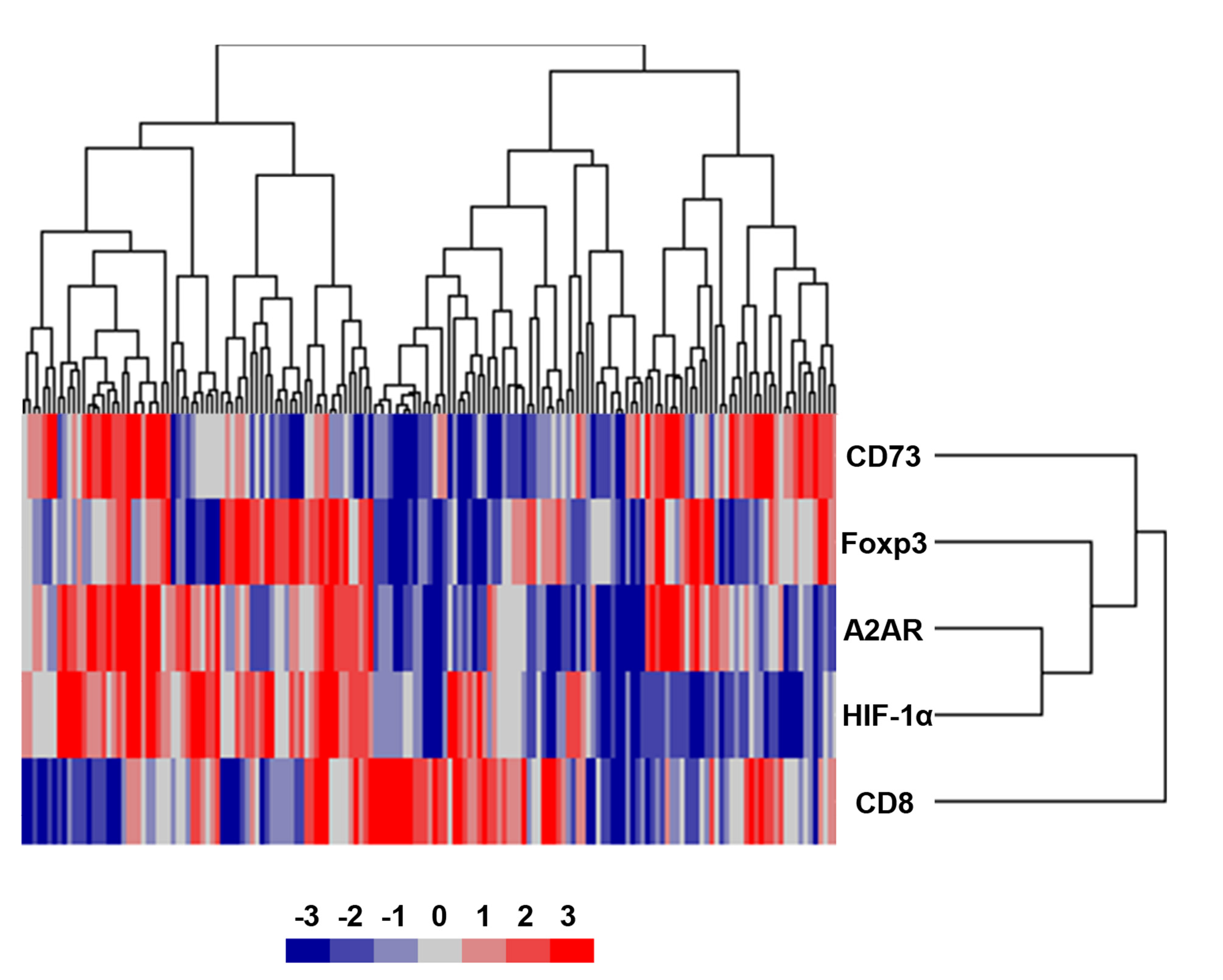

Supplement: Supplementary file 3 — Hierarchical clustering of HIF-1α, CD73, A2AR, CD8 and Foxp3 histoscore in primary HNSCC. The relationship among HIF-1α, CD73, A2AR, CD8 and Foxp3 was determined by Hierarchal clustering analysis in primary HNSCC (n = 165) (JPEG 404 kb) [file 12943_2017_665_MOESM3_ESM.jpg]

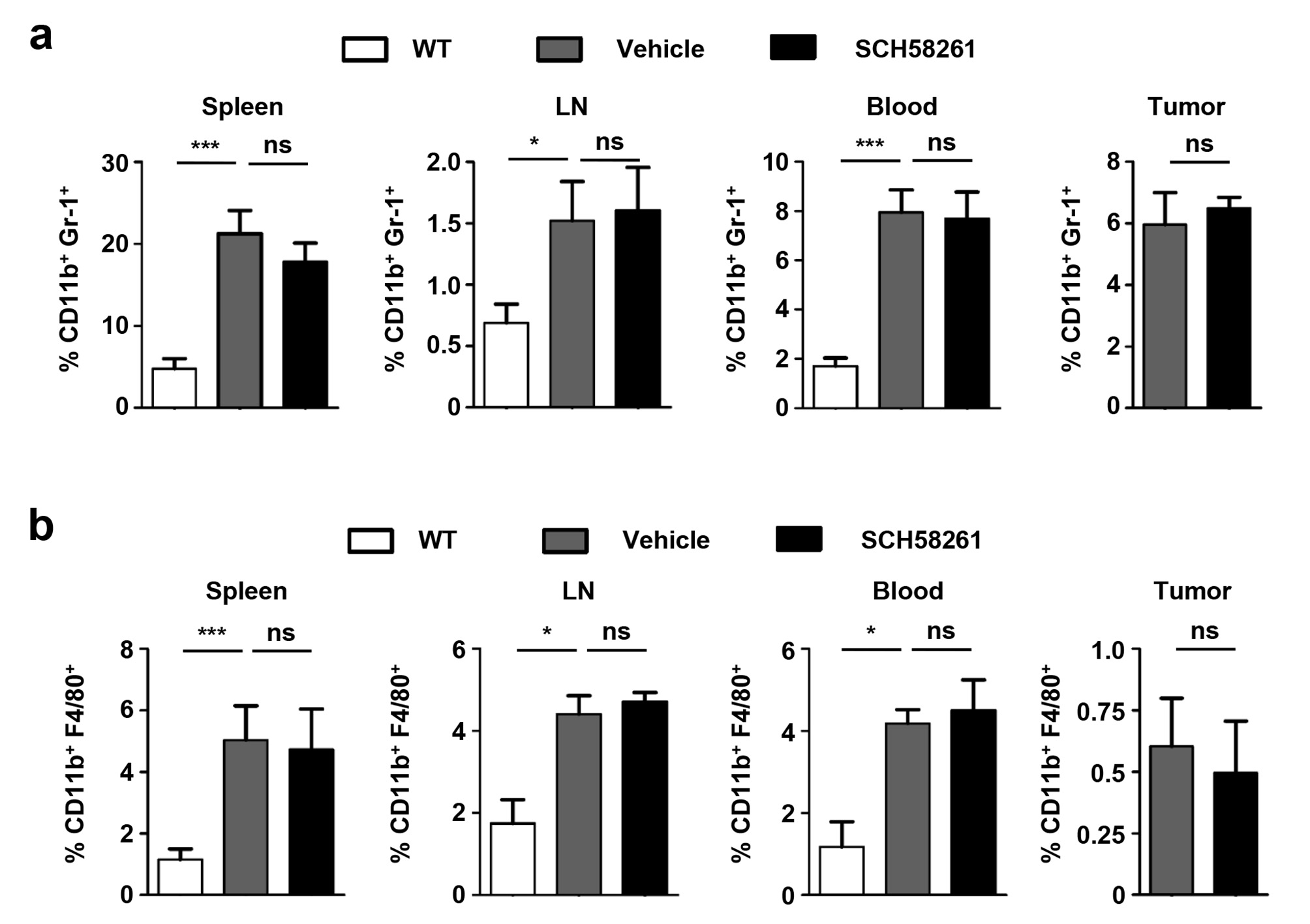

Supplement: Supplementary file 4 — A2AR blockade is unable to influence the population of MDSCs and TAMs. a Quantification of MDSCs (CD11b+ Gr1+) in spleen, lymph node (LN), peripheral blood and tumor from wild type mice (WT) and 2cKO tumor bearing mice treated with DMSO (vehicle group) or with SCH58261(each group n = 6, Mean ± SEM, *, P < 0.05, ***, P < 0.001, ns = no significance, one way ANOVA with post Tukey test). b Quantification of TAMs (CD11b+ F4/80+) in spleen, lymph node (LN), peripheral blood and tumor from WT mice and 2cKO tumor bearing mice treated with DMSO or with SCH58261 (Mean ± SEM, *, P < 0.05, ***, P < 0.001, ns = no significance, one way ANOVA post Tukey test) (JPEG 288 kb) [file 12943_2017_665_MOESM4_ESM.jpg]
